# Animal re-identification in video through track clustering

**DOI:** 10.1007/s10044-025-01497-8

**Published:** 2025-06-19

**Authors:** Francis J. Williams, Samuel L. Hennessey, Ludmila I. Kuncheva

**Affiliations:** School of Computer Science and Engineering, Bangor University, Dean Street, Bangor, Gwynedd LL57 1UT UK

**Keywords:** Animal monitoring, Constrained clustering, Video-based identification, Unsupervised learning

## Abstract

Monitoring a group of animals would greatly benefit from automated animal re-identification from video. Multiple Object Tracking alone does not provide a sufficiently good re-identification, hence we propose to augment the process by further clustering the output tracks. Unlike datasets for person and vehicle identification, existing animal datasets are not substantial enough to train an advanced model for conventional clustering. In this paper, we present a Classification-Based Clustering method (CBC) which employs track labels and temporal constraints to train a bespoke model for each video dataset. Our proposed method works better than using the tracks alone as animal identities. It also outperforms 13 alternative clustering methods applied to the tracking results.

## Introduction

Automated animal re-identification (Re-ID) from video enables conservationists and agriculturalists to monitor their animals without physical implements or cumbersome manual data analysis. Tracking of animals in video is not a novel area [1, 2]; however, individual animal Re-ID is lacking compared to progress in person and vehicle tracking [3, 4]. While general species identification can be useful for some applications, for conservation or agricultural purposes the recent activities of an *individual* animal might be needed. Currently, such tracking is performed using physical tagging or in-person observation which is intrusive to the animals and might lead to a change in their behaviour [5].

To avoid confusion, here we define *animal re-identification in video* to be the recognition of an animal’s identity in any frame of the video containing that animal. The image can come from adjacent frames (forming a tracklet) or from frames further on in the video. Here we do not include videos from concurrently running cameras (cross-camera setting) [6], images taken from a video shot in a different setting, or images from time-lapse camera traps [7]. Our goal is to be able to separate the animal identities in a short video so that we have a “labelled map” of the video. Usually, the animals in the videos we consider are of the same species. A labelled map of a video will aid studies in animal behaviour offering answers to questions such as: which animals like to stick together or avoid one another, whether there there are groups or cliques, who are the solitary individuals, and so on.

Mainstream Multiple Object Tracking (MOT) aims at solving the entire tracking problem, which typically includes bounding box (BB) detection and identification of tracks in a video. Ideally, the tracks found by the MOT algorithm will correspond to the identities in the video. In reality, multiple tracks can be associated with the same identity because the object may leave the camera view and reenter later. Tracks can be broken or misidentified due to occlusion of BBs within a frame. If we assume that each track corresponds to a single animal, it makes sense to *post-process the tracks* in order to join the tracks of the same animal together. This will provide a labelled map of the video. Tracks can be joined together, for example, by a clustering algorithm, where each track is represented in the feature space as the centroid of the object representations within that track. Clustering the tracks will amount to clustering the centroids [8–10]. Thus far, MOT methods produce the tracks based on time and space contingency but do not explore Re-ID possibility by joining tracks into a given number of identities. In this paper, we propose a method to solve this problem by post-processing tracks produced by MOT methods. To this end, we propose to extract features from each BB, thereby creating an unlabelled dataset. Our method is a *classifier-based clustering (CBC)* of the data while using the track labels provided by the MOT algorithm together with the constraints coming from the video. The constraints reflect the fact that any pair of objects coming from the same frame in the video cannot be the same identity.

**Fig. 1:**
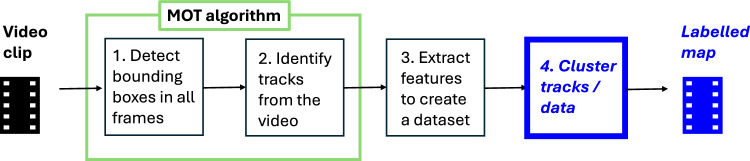
Pipeline of the proposed methodology for creating a label map of a video. The novel elements are indicated in bold italic

The pipeline of the proposed methodology is shown in Fig. 1.

The datasets used in this paper are particularly challenging because they are taken with a hand-held camera, from an angle where the animals can move in front of one another or clump together, obscuring the identifying visual features. A typical lab environment or a stable-based footage would have a static camera mounted at the top, so that the animals are all visible within a confined enclosure [11, 12]. In our video clips, animals move in and out of camera view. Hence, our datasets present significant challenges for object tracking and re-identification, particularly dealing with occlusion and substantial concept drift. Such type of annotated datasets are scarce. In this study, we use all accessible datasets. Other collections exist where typically there is just one animal in a frame which makes the problem of re-ID irrelevant.

We would like to eliminate uncertainties related to the choice of an object detection method [13] and a feature extraction method [14]. These are not part of the proposed CBC. However, we assume that we know, at least approximately, the number of identities to look for in the video.

**Fig. 2:**
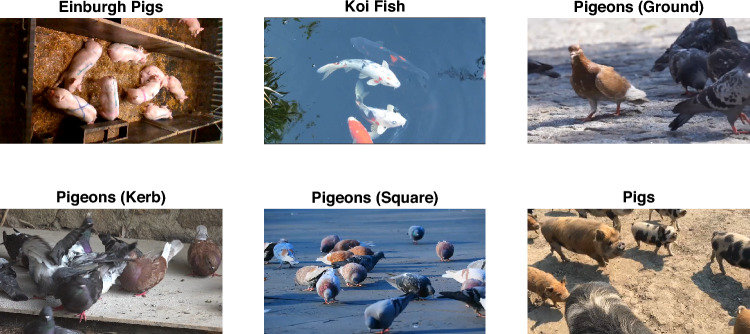
Sample frames from each video type. The Edinburgh Pigs have the same setup in all videos, we thus show one example from that subset

To evaluate the merit of the proposed CBC method, we carry out experiments with 15 video data sets. Arguably, there is a shortage of annotated video clips of the type we are considering here. Five videos have been annotated and publicised by a team including the authors [14, 15]. These videos contain footage of animals in different states and surroundings, e.g.: busy settings with overlap, from stationary and moving, and top-down and side views. Figure 2 shows a sample of the data used.

The rest of the paper is organised as follows. Section 2 contains a review of current literature on MOT and recent advances in track clustering methods. Section 3 outlines the methodology used in the experiment, including a description of our proposed method in Sect. 3.3. In Sect. 4, we propose the pipeline of our experiment. In Sect. 4.1, we detail the dataset used in the study. Finally, we discuss our findings in Sect. 5.

## Related work

### Multiple object tracking (MOT)

MOT is a crucial computer vision task with widespread applications in video surveillance [16–18], autonomous driving [19], and robotics [20]. The primary aim of MOT is to detect, follow, and maintain the identity of multiple objects as they move across sequential video frames to produce tracks, where each track belongs to a single identity. MOT algorithms need to address challenges like occlusions, interactions, and the varying appearances or movement patterns of each object. Over the years, MOT research has evolved from traditional, model-based tracking approaches [21] to deep learning-powered models that leverage advancements in object detection, appearance modelling, and data association [22].

The shift to deep learning-based object detection marked a significant milestone in MOT. Detectors like YOLO, Faster R-CNN, and SSD substantially improved the accuracy and speed of object detection, forming the basis for more robust MOT systems. These detectors provided more reliable BBs and object features, which greatly enhanced object association across frames. Deep learning also enabled appearance-based feature extraction, allowing for more precise identity maintenance in crowded scenes [23].

A significant challenge in MOT is re-identification (Re-ID)  [24–29]. Traditional interpretation of Re-ID is identification of an object from concurrently running cameras. In our study, we interpret Re-ID as recognising the same object in different parts of the video, possibly far from one another in time. Tracking, as well as traditional Re-ID usually do not solve this problem entirely. MOT algorithms often produce multiple tracks for a single object. The challenge is to cluster these tracks into coherent groups that accurately represent the identity of the objects. MOT algorithms can cope with temporary interruptions in the object’s visibility. They construct tracklets which are eventually combined into tracks [30–32]. We argue that the tracks themselves can be further combined in a quest to get a better match of the object identities while disregarding their temporal component.

### Animal re-identification

While the integration of deep learning and advanced feature extraction has greatly enhanced MOT systems, there are certain scenarios where these models may not be practical. An example of this would be animal Re-ID. The vast majority of current contributions in the field of MOT systems are targeted towards person Re-ID [24, 33]. Researchers are continually developing more complex and robust methods to distinguish people’s identities. Brown et al. [34] propose the Multi-Modal High Precision Clustering method that takes advantage of face tracks, body tracks and voice tracks together to achieve more accurate person Re-ID. There are methods which track multiple animals in a video, especially with respect to activity monitoring and pose estimation [35–38]. A limitation of these methods is they do not label the animals that are found.

The main challenge in animal Re-ID is the limited availability of data to train deep learning models, especially when each application involves different animals, potentially from entirely different species. Zuerl et al. [39] provide an extensive video database for the identification of polar bears. 13 animals were filmed *separately* and the video clips were used to learn the appearance and the movement of each bear. While this is a precious resource for the animal Re-ID research community, we cannot use it here. Our task is Re-ID of multiple animals appearing *together* in the same frame.

A highly successful algorithm for tracking animals such as zebra fish, mice and fruit files has been developed by Perez et al. [40]. Their videos are from a static camera positioned above the enclosure with the animals, so that trajectories can be inferred primarily by position and motion clues. Such tracking algorithms will not work on our dataset because (1) we do not use a static, top-view camera, (2) the animals in our videos may go out and in camera view, and (3) There is substantial occlusion of BBs.

Zhang et al. [41] report excellent accuracy in pig Re-ID from video feed. They use a top-view static camera which covers the whole pen, so all animals are in view at any time. This makes the tracking task more straightforward compared to tracking from unrestricted video. The occlusion problem in their study is caused by an insect occluding the camera rather than objects occluding one another.

Tracking blackbuck antelopes from video footage of concurrently running UAVs (drones) has been proposed by Naik et al. [42]. Their extensive database contains annotated videos that are suitable for tracking and monitoring long-term behaviour of the animals. The Re-ID in this case is meant to fuse data in the overlap between two adjacent video streams coming from the two drones. While highly valuable as a research resource, this dataset is different from our target. In the aerial images, the animals are clearly identifiable, with no occlusion. However, the BB overall size is relatively small, which makes it difficult to extract features and train a classifier to recognise the individual animal. In our envisaged applications, the BBs are large and overlapping because of the position of the camera.

For the purposes of our study, we can assume that tracking has been completed using the best state-of-the-art algorithm. We argue that clustering the resulting tracks using the feature representation of the participating objects will lead to improved recognition accuracy.

## Methodology

### Preliminaries

Let $F =\{f_1,\ldots,f_M\}$ be a set of consecutive video frames comprising the video clip. Consider each $f_i$ to be an RGB image of a size determined by the camera resolution. A MOT algorithm applied on *F* will return a set of BBs for each frame, as well as a track label for each BB. Let $Z = \{z_1,\ldots,z_N\}$ be the set of BBs identified by the MOT algorithm. For each $z_i$, the MOT algorithm will return

- (a) Frame number $$ j = Fr(z_i)$$
- (b) BB’s coordinates in the frame $$ [x,y,w,h] = Co(z_i)$$
- (c) Track label $$ k = Tr(z_i)$$.
  Additionally, we assume that we have access to a feature extractor (a simple colour space or deep NN features), that is,
- (d) Feature vector $\textbf{x}_i = Fe(z_i)$. The feature vector can be obtained from any source, for example, Autoencoder, deep NN, HOG, etc.

The task is to assign identity labels to all elements of *Z*, assuming that we know, at least approximately the number of true identities in the video.

We regard animal re-ID as a label-matching task. Each BB has a ground truth label $l= Gt(z_i)$. The task is to use the information contained in *k*, $\textbf{x}_i$, and *j* to derive a proposed label for $z_i$. Typically, in a standard classification task, only $\textbf{x}_i$ would be used to train a classifier. In the scenario considered here, we have extra contextual information. Our proposed CBC method aims at combining all sources of information in order to create *l*.

To apply the proposed CBC method, we use (a), (c) and (d). We assume that the information contained in (b) has already been used by MOT for finding the tracks. To verify that there is merit to the track post-processing, we shall assume that we have ground truth labels.

The metric of choice for comparing the ground truth and the obtained labels is the Hubert-Arabie Adjusted Rand index, discussed in the next subsection.

There are several possible approaches to this problem:

A. Do not post-cluster the tracks. Use the track labels (c) as the obtained labels. We include this approach for the following reason. If the tracks are pure, which means that each track corresponds to a single identity, track combination may be beneficial. If, however, tracks contain mixed identity (highly likely with our type of data), combining may be futile.
B. Use an off-the-shelf clustering method to cluster the raw data (d), ignoring the track labels.
C. Use a constrained clustering method on (d) where the tracks (c) define the ML constraints, and the CL constraints are derived from the frame incidences (a).
D. Find the centroids (d’) of the tracks (d) and treat them as a new data set. Cluster these centroids using off-the-shelf clustering methods.
E. As the tracks already include the Must-Link (ML) constraints, find the Cannot-Link (CL) constraints between *tracks* using (a) and apply a constrained clustering method to the centroids (d’).
F. Use the track labels (c), the frame incidences (a) and the data (d), without reducing the tracks down to a set of centroids.

Our CBC method is based on the latter approach.

### Metrics

This subsection explains our choice of metric. Albeit a detour from the main gist of the paper, we believe this to be an important argument. We considered a standard collection of metrics used in the literature on clustering: Normalised Mutual Information (NMI) [43], Adjusted Rand Index (ARI) [44], Counting Accuracy (ACC) and Hungarian Algorithm Accuracy (HACC) [45]. The metrics compare two column vectors containing the True Labels (TL) and the Assigned Labels (AL). *NMI*(*TL*, *AL*), *ACC*(*TL*, *AL*), and *HACC*(*TL*, *AL*) all return a value between 0 and 1, where 0 signifies no relationship between TL and AL and 1 signifies perfect relationship. *ARI*(*TL*, *AL*) returns a value between $-1$ and 1, where 1 indicates a high agreement between the labelling, 0 implies random labelling, and $-1$ indicates high disagreement. We do not consider HOTA/MOTA metrics for tracking as we use ground truth BBs in this study, therefore BB correctness is already guaranteed.

To illustrate the point, we took one of the datasets (EP000036 from the Edinburgh Pigs dataset) in our experiment. It contains eight pigs in a pen, i.e., eight true identities, $N=699$ objects, and $K=463$ tracks. If we are to measure the success of clustering the tracks it stands to reason to compare the result with the pre-clustered track labelling. The true labels (TL), or ground truth, are the ones provided in the annotated dataset, and the assigned labels (AL) are the track labels. In order to verify that the metrics truly represent the quality of the clustering, we devise four additional labellings of the objects.

- RL. Random Labels. We generated *N* random labels in 8 classes (*N* random integers from 1 to 8).
- SL. Same Labels. Assume that the algorithm assigns the same class label to all objects. Here we generated label 1 for all the objects.
- DL. Different Labels. Here we assume that each object has a different label. We generated a random permutation of all the integers from 1 to *N*.
- RA. Random Assigned Labels. As there are 463 tracks, we generated *N* random integers from 1 to 463, which randomly assigns each object to a track.

The values of the metrics comparing TL with the four generated label sets are shown in table Table 1.

**Table 1:** Comparison of TL with the four generated label sets

Labeling	NMI	ARI	ACC	HACC
AL	0.2353	0.0557	0.4750	0.2089
RL	0.0151	$-$0.0009	0.2732	0.1788
SL	0.0000	0.0000	0.2589	0.2589
DL	0.5367	0.0000	1.0000	0.0114
RA	0.1435	0.0000	0.3634	0.0529

A reasonable metric is expected to find a value of 0 for the generated labels and a non-zero value for the assigned labels (the genuine tracks returned by the tracking algorithm). As the table shows, NMI as well as the two classification accuracy metrics score some of the additional labellings higher than AL which is unacceptable. This leaves only the ARI as a suitable metric for the experiment with the type of datasets we consider here, where the number of clusters in AL is much different to the number of clusters in TL. Therefore, we will be using ARI in the rest of the paper.

### Classifier-based clustering (CBC)

For approaches B and D, we use readily available clustering methods as built into MATLAB. These are:

1. k-means
2. Average Linkage
3. Centroid Linkage
4. Complete Linkage
5. Median Linkage
6. Single Linkage
7. Ward Linkage
8. Weighted Linkage
9. FINCH. Here we decided to include the FINCH algorithm (First Integer Neighbour Clustering Hierarchy) [46] which has been found to be successful in people re-identification [34]. We have used this method with the default parameters found within the MATLAB implementation presented by the authors.
10. Gaussian Mixture Models. We used a diagonal shared covariance matrices with maximum iteration count of 1000.
11. DBSCAN. For this method we found that the choice of maximum distance parameter ($\epsilon$) effects the clustering results dramatically. Therefore, we run DBSCAN with $\epsilon \in \{0.5, 1.0, \ldots, 4.0\}$. MinPts is another DBSCAN parameter which specifies the number of points required for a dense region. For every value of $\epsilon$, we run DBSCAN with MinPts $\in \{1,2 \ldots, 8\}$. Finally, we picked combination of $\epsilon$ and MinPts that gave us the number of clusters closest to the desired number.
12. Spectral Clustering.

For approaches C and E, we use a Constrained Clustering Ensemble method (CCEN) [47], which proved to be the best option for our type of data. CCEN incorporates temporal pairwise ML and CL constraints. We found the optimum parameters for this method to be an ensemble size of 5, using average linkage as the base clusterer.

The idea of the proposed CBC method is to make use of the track labels without collapsing (and thereby over-simplifying) the tracks into centroids. In addition, we take into account the CL constraints between the tracks. A classifier is trained on the data with the current track labels, and a resubstitution confusion matrix *M* is calculated. Let *p* and *q* be two different tracks (classes). If *M*(*p*, *q*) is large, the trained classifier has mistaken many BB’s belonging to track *p* as those belonging to track *q*. This would suggest that *p* and *q* may be the same identity. However, instead of the exact number of mislabellings, we are interested in the proportion of track *p* being labelled as track *q*. A large proportion would suggest the same identity. Based on the value within the scaled confusion matrix *M*, our algorithm merges tracks *p* and *q*.

Notice that the classifier uses only the feature data in its training, which means that the objects are classified by similarity/appearance, while disregarding spatio-temporal connections.

The proposed algorithm is ‘monolithic’, in that it is based on a single idea: constrained clustering through classification. The only element that seems to be suitable for eliminating for an ablation study is the scaling of the confusion matrix *M*. We did try this in a pilot experiment, but the results were significantly worse.

Using the notation introduced above, the steps of the method are as follows:

**Table:** 

Classifier-Based Clustering Algorithm (CBC)	
**Input:** A dataset $X = \{\textbf{x}_1,\ldots,\textbf{x}_N\} = Fe(Z)$, a frameset $Y = \{y_1,\ldots,y_N\} = Fr(Z)$, a track-label set $L = \{l_1,\ldots,l_N\} = Tr(Z)$, and a desired number of clusters *K*. (It is assumed that *K* is smaller than the number of tracks. If not, the track labels should be used directly.)	
**Initialisation:** Choose a classifier model *C*. Create a set $L'\leftarrow L$ of current track labels.	
1. Train *C* on the data set *X* using the current track labels $L'$.	
2. Relabel all data in *X* using *C* (resubstitution) and create a confusion matrix *M*.	
3. Set the main diagonal of *M* to 0, as we are not interested in the correct classifications at this stage.	
4. Using the frameset *Y* and the current track label set $L'$, identify CL constraints between pairs of tracks. For each CL constraint (*p*, *q*), set $M(p,q)=M(q,p)=0$. This step is needed to make it impossible to join tracks bounded by a CL constraint.	
5. Scale each row of *M* to sum up to 1. Thus, entry *M*(*i*, *j*) of *M* will be the proportion of current track *i* labelled as current track *j*. A large proportion will indicate that these two tracks may be representing the same identity.	
6. Identify the largest element of *M*.	
(a) If that value is zero (nothing available to merge) or the number of current tracks is equal to *K*, exit the algorithm and return the current labels $L'$.	
(b) Else, combine the tracks of the row and the column by relabelling all points of the row index in $L'$ to the track corresponding to the column index. Continue from Step 1.	
**Output:** The set $L'$ of current track labels.	

Below is a list of properties of CBC:

- *Correctness.* Functional algorithm correctness ensures that for each input the algorithm produces the expected output, according to a given specification. For the proposed CBC algorithm, the functional correctness is guaranteed by design. The algorithm will always return a set of cluster labels for the input dataset. Starting with the initial track label set, the only change to these will occur when two clusters are merged, and the labels are reassigned at step 6b. Thus, each object will receive a legitimate cluster label.
- *Completeness.* The completeness of an algorithm ensures that it does not miss any possible input. CBC is complete by design because it will work with any labelled input data (in this case, the labels are defined by the tracks).
- *Convergence.* The convergence of the CBC algorithm is guaranteed by the fact that there are a limited number of initial track labels, say, *M*. The number of desired clusters *K* is smaller than *M*. If all merges are possible (there are no CL constraints), the algorithm will stop after $M-K$ steps. If there are CL constraints, the steps will be fewer.
- *Time complexity.* The time complexity of the algorithm is largely dependent on the classifier used at step 1. The maximum possible number of steps is $M-1$, when $K=1$ and there are no CL constraints. We noted that, due to the classifier training, CBC takes longer than the standard clustering algorithms. Notice that the proposed algorithm is open to any classifier model. While definitely an important aspect, efficiency is not of prime concern at this stage because the algorithm is meant to work off-line (post-processing).

## Experiment

### Data

#### General description of the dataset

The dataset used in our experiment consists of 15 video clips containing multiple animals. Each clip contains animals of the same species. Ten videos are obtained from the Edinburgh Pig Behaviour Video Dataset [48], and the remaining five are from our bespoke Animal Video Dataset [14].

The videos exhibit a variety of obstacles to the MOT problem. These include: moving and stationary cameras; top-down as well as side-on views of the animals; various degrees of occlusion between animals; and difficult-to-distinguish individuals. In the Edinburgh Pig videos, the animals are bounded within the camera frame by fencing, whereas in the remainder of the videos the animals are free to exit and re-enter the field of view of the camera.

The animals in the video were manually annotated with BBs. For each BB, we extract 54 RGB features as described in [45]. In short, each BB is separated into a 3-by-3 grid and from each cell, the mean $\mu$ and the standard deviation $\sigma$ of each colour plane (R/G/B) is extracted, giving 9 cells $\times$ 3 colours $\times$ 2 ($\mu$ and $\sigma$) = 54 features. Through extensive experiments in the past, we ascertained that these features were best suited to our data, compared to deep features (CNN), features obtained from an autoencoder, as well as HOG, LBP, and hue histogram features [49]. The reason behind this finding is that the BBs in our data are highly overlapping, often containing more than one animal, and that some of the animals are quite similar in appearance. We argue that the simplest feature extractor works best because of the complexity of the data. If the clusters had a clear but complex spatial distribution, a sophisticated model such as deep learning would be a good choice. In our case, the clusters (identities) are not clearly distinguishable even by human eye. Much of the annotation was done by the relative position of the animal in the video frame rather than by the appearance of the bounding box.

**Fig. 3:**
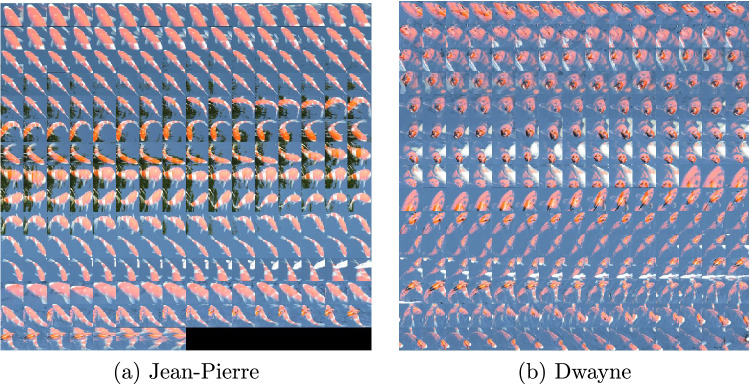
Class Jean–Pierre and class Dwayne from the Koi fish video

To demonstrate the difficulty of the problem, and specifically the intra-class variability of the data, we prepared a collection of all BBs belonging to class Jean-Pierre and all BBs belonging to class Dwayne from the Koi fish video (Fig. 3). The appearance of both fish varies considerably throughout the video. Besides, there are significant similarities between the two identities.

#### Track generation

A tracking algorithm typically includes two steps: object detection and association [50, 51]. For each frame in the video, the object detection step returns a set of BBs within a frame, and the association step links the BBs in consecutive frames into tracklets. We decided to bypass the object detection step by supplying the true BBs in each frame to the tracking algorithm. This means that the tracking algorithms will perform only the association step. The true BBs are the ones created by the manual annotation of the videos. In doing so, we eliminate potential problems related to object detection. Our goal is to examine how well the tracks obtained under most favourable circumstances can be used in animal Re-ID.

For the purpose of our experiment, we have generated three sets of track files for each video.

- *MATLAB* The first is a standard MATLAB tracking algorithm created using the Automated Driving Toolbox. The ground truth BBs were inserted in the algorithm in the place of the BBs determined by the algorithm itself. Tracks are generated by the MOT algorithm by predicting the BB position in the next frame through Kalman filter and assessing the suitability of the newly detected BBs using Global Nearest Neighbour.^1^ The appearance of the BB has no influence on the track generation. Collision of tracks is resolved by following the trajectory.
- *BASIC* The second set of tracks was based on temporal ML constraints. The idea is to combine BBs in adjacent frames where the intersection over union (IoU) of the two boxes exceeds a predefined threshold. In our experiment, we set the threshold to 0.7, i.e. a 70% overlap between the boxes. The track generation algorithm takes each pair of consecutive frames and calculates the IoU between all pairs of boxes, one from each frame. The Munkres algorithm is then applied to assign each box to its best fit in the adjacent frame. This step is particularly relevant in the case of overlap where two objects in one of the frames might compete to be matched with the same object in the other frame.
- *FCG* The third method for creating tracks was Feature Combinatorial Grouping (FCG) [31], which operates on the principle that instances of the same object exhibit similar appearance traits within a small temporal range. FCG is structured in two main stages: initially, it generates a set of short tracklets, and then, in the second stage, it sequentially merges these tracklets over time using a hierarchical clustering process guided by “lifted frames”. A lifted frame represents an artificial time interval grouping multiple tracklets instead of individual object detections. This clustering method uses UPGMA (Unweighted Pair Group Method with Arithmetic Mean) to iteratively combine cluster pairs, ultimately forming a hierarchical structure that produces the final set of object tracks. We were able to integrate our RGB feature representations into the FCG algorithm, enabling a fair comparison.

### Experimental protocol

The purpose of this experiment is to examine the six approaches from Sect. 3.1 for animal Re-ID. As such, we split the experiment into sections accordingly:

The experiment follows the notation A-F for the six approaches, as introduced above. The following list gives more details for each approach:

$$\textcircled {A}$$
The track labels from the three tracking methods *MATLAB*, *BASIC* and *FCG* for each video are compared to the Ground Truth labels directly. (3 results for each dataset)
$$\textcircled {B}$$
Twelve clustering methods were applied to the raw data as shown in Sect. 3.3. (12 results)
$$\textcircled {C}$$
To apply a constrained clustering method to the raw data, we can use ML constraints as well as CL constraints. The CL constraints come from each frame. The ML constraints can be generated by the tracks from *MATLAB*, *BASIC* and *FCG*, respectively. The constraint clustering method chosen for this task was CCEN. (3 results)
$$\textcircled {D}$$
For this part, we calculated the centroids (d’) of the tracks (d) and treated them as a new data set. We applied the 12 clustering methods listed above to each set of tracks. ($12\times 3 = 36$ results)
$$\textcircled {E}$$
CCEN was applied to the datasets where the items being clustered were the track centroids. (3 results)
$$\textcircled {F}$$
CBC was applied to the raw data taking as initial class labels the tracks from *MATLAB*, *BASIC* and *FCG*, respectively (3 results).

Therefore, there were 60 results for each dataset where a set of assigned cluster labels was compared to the true labels through ARI.

The MATLAB code for this experiment is available at https://github.com/frankmnb/Animal-ReIDentification-in-Video-through-Track-Clustering.

## Results

Table 3 (given in Appendix A) shows the ARI values obtained in the experiment. From Table 3, we prepared Table 2 where the 60 methods are arranged by rank, from best (smallest) to worst. The best performing method is our proposed CBC with Basic Tracks. However, there is no single method which is universally best for our data. We apply the Friedman test *incrementally* to find out which group of methods is significantly better than the rest. First, we compare the two best methods. Subsequently, we add one method at a time and calculate the *p*-value of the hypothesis that the methods in the group are indistinguishable. The cut-off point of $p<0.05$ was chosen to measure which group of methods from the top of the table are indistinguishable. This point is marked by a horizontal line in Table 2.

**Table 2:** Friedman test on all methods from our proposed approaches

**Method**	**ARI**	**Rank**	**p-value**
(F) CBC BASIC	0.2449	10.8000	–
(C) Raw-CCEN BASIC	0.2286	11.9667	0.1967
(D) BASIC-Kmeans	0.2049	12.3667	0.5488
(D) BASIC-FINCH	0.1864	12.8667	0.5641
(E) CCEN BASIC	0.2283	14.1000	0.4873
(D) BASIC-Ward linkage	0.1838	14.1667	0.4159
(B) Raw-Ward linkage	0.1586	14.2667	0.6483
(B) Raw-GMM	0.1545	14.4667	0.8166
(B) Raw-Kmeans	0.1699	14.8000	0.8815
(B) Raw-FINCH	0.1563	15.6667	0.8991
(D) BASIC-GMM	0.1283	16.8333	0.7936
(A) Tracks-Only FCG	0.1575	19.9333	0.5244
(D) BASIC-Complete linkage	0.1686	20.1667	0.1328
(B) Raw-Complete linkage	0.1346	20.3333	0.0169
(B) Raw-Weighted linkage	0.1406	21.9000	0.0012
(F) CBC FCG	0.1538	22.3333	0.0011
(E) CCEN FCG	0.1551	22.5000	0.0011
(A) Tracks-Only MATLAB	0.0870	22.6667	0.0010
(C) Raw-CCEN FCG	0.1552	22.7000	0.0010
(C) Raw-CCEN MATLAB	0.1209	22.7667	0.0003
(F) CBC MATLAB	0.1194	24.2667	0.0001
(D) BASIC-Weighted linkage	0.1439	25.1000	0
(E) CCEN MATLAB	0.1158	25.1000	0
(A) Tracks-Only BASIC	0.1313	25.1333	0
(D) FCG-Complete linkage	0.1416	27.4333	0
(D) FCG-Ward linkage	0.1418	27.5000	0
(D) FCG-Spectral	0.1415	27.9000	0
(D) FCG-Weighted linkage	0.1399	27.9333	0
(D) FCG-Kmeans	0.1383	28.2333	0
(D) FCG-Average linkage	0.1397	28.6667	0
(D) MATLAB-FINCH	0.0751	29.0000	0
(D) FCG-DBSCAN	0.1369	30.1333	0
(D) FCG-Centroid linkage	0.1386	30.6333	0
(D) FCG-Median linkage	0.1386	30.7667	0
(B) Raw-Average linkage	0.1122	31.1667	0
(D) FCG-Single linkage	0.1380	31.3333	0
(D) BASIC-Spectral	0.1410	32.3667	0
(D) FCG-FINCH	0.1132	33.8000	0
(D) MATLAB-Ward linkage	0.0619	34.8333	0
(D) MATLAB-Kmeans	0.0914	35.7333	0
(D) BASIC-Average linkage	0.1140	37.3667	0
(D) BASIC-DBSCAN	0.0612	37.8667	0
(D) MATLAB-Complete linkage	0.0505	39.5000	0
(D) MATLAB-Spectral	0.0821	40.1333	0
(D) BASIC-Median linkage	0.0940	40.1667	0
(B) Raw-Spectral	0.0767	40.2000	0
(D) MATLAB-Weighted linkage	0.0785	41.2667	0
(B) Raw-Median linkage	0.0770	41.3000	0
(D) MATLAB-GMM	0.0185	44.0667	0
(D) BASIC-Centroid linkage	0.0806	46.1000	0
(D) MATLAB-Average linkage	0.0705	46.2667	0
(D) MATLAB-DBSCAN	0.0368	46.7667	0
(B) Raw-Centroid linkage	0.0633	47.3000	0
(D) MATLAB-Centroid linkage	0.0666	48.3667	0
(D) BASIC-Single linkage	0.0748	48.4333	0
(D) MATLAB-Median linkage	0.0670	48.5000	0
(B) Raw-Single linkage	0.0798	48.5667	0
(D) FCG-GMM	0.0789	49.6333	0
(D) MATLAB-Single linkage	0.0543	51.2333	0
(B) Raw-DBSCAN	0.0037	52.3333	0

**Fig. 4:**
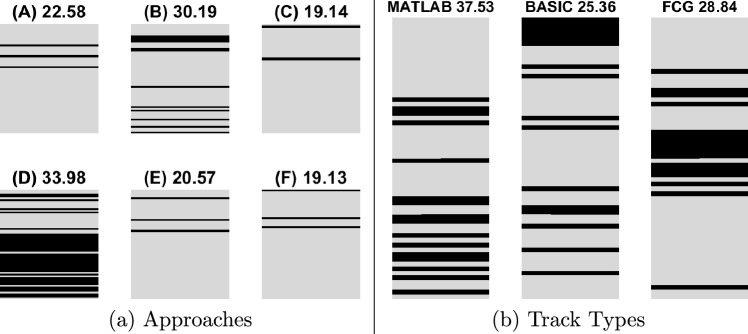
Position of the category in the ranking table. The higher the position, the better the category against the alternative. The average rank for the category is also shown

Figure 4 depicts visually the efficacy of the sixty methods on our datasets. Table 2 is represented as a grey block. For each block, a black stripe is added where the keyword in the label is found. Subplot (a) shows the positions of the 6 approaches. The title above each block shows the average rank for the respective approach (the smaller, the better). Subplot (b) shows the results for the track type.

Based on Table 2 and Fig. 4 we make the following observations

- The proposed CBC method (F) shows the best overall result with the *BASIC* tracks. The advantage of CBC is only marginal, with constrained clustering of the raw data (C) closely following.
- There is no clear winner, either as a single method or an approach. The CBC approach (F) has an overall rank that is marginally better than the constrained clustering of raw data (C).
- Non-constrained clustering of track centroids (D) is, in fact, worse than using the tracks-only (A). This goes against the popular and intuitive rhetoric of current research. We attribute this result to the inherent structure of our datasets which prevents using more advanced feature extraction methods, e.g., deep features.
- Constrained clustering (F), (C) and (E) improves on the tracks-only (A), proving our main point. Hence, constrained post-clustering of the tracks is what we would recommend in future work.
- It turns out that advanced tracking methods may not work well on this type of data. The simple *BASIC* tracks method based on IoU match is sufficiently effective.

## Conclusion

In this paper, we introduce a Classifier-Based Clustering (CBC) method for individual animal Re-ID from video. Our findings demonstrate that applying additional processing to tracking data yields better results than using the tracks alone, underscoring the need for a post-processing method like CBC. Although our method shows only a slight improvement over constrained clustering on raw data, further comparisons with larger and more varied datasets could reveal clearer distinctions between CBC and raw data clustering. Our experimental analysis confirms that MOT tracks alone do not produce the optimal label map for animal Re-ID from video, highlighting the importance of developing post-processing techniques within this research area.

However, some limitations should be noted. Identifying animals in a short video may not generalise well in recognising the same animals in different videos or images, particularly if camera settings vary. Additionally, having a labelled map for a video does not imply the existence of a universal classifier applicable to any image. Even under consistent camera settings, changes in lighting and other conditions may render any classifier model less effective.

Currently, CBC takes substantially longer to run compared to the alternatives in our experiment. In future works, we will aim to reduce the CBC running time. This will be of particular interest should larger annotated datasets of the type considered here become available.

Finally, an interesting continuation of our work is to cluster the tracks as they come in the video using CBC or another constrained clustering approach. This will partly reduce the noise in the data, as the tracks are likely to return compact clusters. Post-clustering a smaller subset of tracks may lead to better overall accuracy.

## Adjusted rand index scores

The 60 rows of Table 3 correspond to the approaches A-F, as explained in Sect. 4.2. The values are given in percentages rounded to the nearest whole number.

**Table 3:** Percentage Adjusted Rand Index scores for all methods as they perform on the video datasets

	EP000002	EP000005	EP000009	EP000010	EP000016	EP000028	EP000033	EP000036	EP000060	EP000078	Koi Fish	Pigeons (Ground)	Pigeons (Kerb)	Pigeons (Square)	Pigs
(A) Tracks-Only MATLAB	10	4	6	6	7	7	5	6	17	6	19	17	5	15	2
(A) Tracks-Only BASIC	5	5	4	3	2	3	2	2	13	2	64	54	9	26	3
(A) Tracks-Only FCG	1	34	1	9	15	22	4	12	91	27	13	5	1	1	1
(B) Raw-Single linkage	0	4	0	0	15	0	0	0	100	0	0	0	0	0	0
(B) Raw-Complete linkage	2	17	2	4	23	9	2	9	49	17	15	9	10	24	10
(B) Raw-Average linkage	0	19	1	0	13	8	0	8	75	16	8	5	1	12	3
(B) Raw-Weighted linkage	1	19	4	3	24	7	1	6	75	10	16	11	6	18	10
(B) Raw-Centroid linkage	0	4	0	0	6	2	0	2	75	0	4	1	1	0	1
(B) Raw-Median linkage	0	6	0	0	19	3	0	1	75	3	1	1	1	4	0
(B) Raw-Ward linkage	4	18	4	5	30	12	4	20	24	18	15	13	16	36	18
(B) Raw-Kmeans	3	20	3	4	28	13	4	14	48	15	18	14	19	35	16
(B) Raw-GMM	3	22	4	6	29	14	5	14	22	17	22	10	16	32	16
(B) Raw-FINCH	3	19	3	3	25	16	3	15	27	17	19	13	18	34	19
(B) Raw-Spectral	0	7	0	1	7	4	0	13	62	9	8	0	0	0	3
(B) Raw-DBSCAN	0	4	0	1	3	0	0	1	−7	3	0	0	0	0	0
(C) Raw-CCEN MATLAB	5	7	4	4	8	7	4	7	84	5	15	11	5	14	2
(C) Raw-CCEN BASIC	2	23	2	5	65	16	6	21	49	24	52	43	8	25	2
(C) Raw-CCEN FCG	0	34	1	8	15	22	4	11	91	27	13	5	1	1	1
(D) MATLAB-Single linkage	0	0	0	0	0	0	0	0	70	0	4	1	0	5	1
(D) MATLAB-Complete linkage	2	5	0	0	1	2	1	−1	35	5	10	3	3	8	2
(D) MATLAB-Average linkage	0	3	0	0	−1	1	0	1	85	0	6	3	0	6	2
(D) MATLAB-Weighted linkage	0	7	0	1	−1	4	0	1	85	3	6	3	1	6	2
(D) MATLAB-Centroid linkage	0	1	0	0	−1	1	0	0	85	0	5	1	0	5	1
(D) MATLAB-Median linkage	0	2	0	0	−1	1	0	1	85	0	5	1	0	5	1
(D) MATLAB-Ward linkage	2	10	0	1	1	6	1	1	35	5	6	11	3	9	2
(D) MATLAB-Kmeans	1	9	0	0	5	7	1	1	85	4	6	4	3	9	2
(D) MATLAB-GMM	1	2	0	1	3	2	0	2	0	5	0	9	2	0	0
(D) MATLAB-FINCH	2	0	8	4	6	7	4	1	31	4	15	17	4	10	1
(D) MATLAB-Spectral	1	1	0	0	0	0	0	0	85	0	6	12	3	13	2
(D) MATLAB-DBSCAN	0	5	0	0	4	4	0	1	28	1	5	1	0	6	0
(D) BASIC-Single linkage	0	4	0	0	4	0	0	0	100	1	0	3	0	0	0
(D) BASIC-Complete linkage	3	17	4	4	24	11	3	14	100	16	23	23	0	11	1
(D) BASIC-Average linkage	1	17	0	0	12	5	0	2	100	17	8	7	0	3	0
(D) BASIC-Weighted linkage	2	19	2	3	12	10	0	16	100	7	23	15	1	6	0
(D) BASIC-Centroid linkage	0	3	0	0	8	0	0	3	100	0	0	6	0	1	0
(D) BASIC-Median linkage	0	7	0	0	11	3	0	3	100	0	7	8	0	1	0
(D) BASIC-Ward linkage	5	16	4	4	25	12	8	20	100	23	22	23	1	12	2
(D) BASIC-Kmeans	5	18	4	5	37	12	7	17	100	14	42	28	4	13	2
(D) BASIC-GMM	4	17	4	6	34	13	5	18	0	20	27	29	1	14	2
(D) BASIC-FINCH	5	19	4	5	26	10	6	18	46	15	47	45	9	21	4
(D) BASIC-Spectral	0	15	0	1	28	9	0	11	100	14	0	27	0	5	1
(D) BASIC-DBSCAN	2	5	0	1	3	0	0	3	38	7	19	10	1	3	0
(D) FCG-Single linkage	0	34	0	0	15	21	1	6	91	23	13	0	1	1	1
(D) FCG-Complete linkage	0	34	1	2	15	21	2	6	91	23	13	2	1	1	1
(D) FCG-Average linkage	0	34	1	0	15	21	1	6	91	23	13	2	1	1	1
(D) FCG-Weighted linkage	0	34	1	0	15	21	1	6	91	23	13	2	1	1	1
(D) FCG-Centroid linkage	0	34	0	0	15	21	1	6	91	23	13	1	1	1	1
(D) FCG-Median linkage	0	34	0	0	15	21	1	6	91	23	13	1	1	1	1
(D) FCG-Ward linkage	0	34	1	2	15	21	2	6	91	23	13	2	1	1	1
(D) FCG-Kmeans	0	31	1	4	15	20	1	6	91	23	13	1	1	1	1
(D) FCG-GMM	0	0	0	0	15	0	0	0	91	0	13	0	0	0	0
(D) FCG-FINCH	0	22	1	4	15	9	1	− 1	91	10	13	3	0	0	1
(D) FCG-Spectral	0	32	1	3	15	21	2	6	91	23	13	4	1	1	1
(D) FCG-DBSCAN	0	33	0	0	15	21	1	6	91	23	13	0	1	1	1
(E) CCEN MATLAB	6	5	5	2	10	5	5	4	84	3	15	10	4	14	2
(E) CCEN BASIC	2	18	2	4	67	15	5	19	49	23	60	45	7	25	2
(E) CCEN FCG	0	34	1	8	15	22	3	11	91	27	13	5	1	1	1
(F) CBC MATLAB	10	6	5	3	11	4	5	7	79	6	12	12	5	14	2
(F) CBC BASIC	10	13	6	6	36	9	6	13	94	11	74	51	9	27	2
(F) CBC FCG	1	33	1	8	15	22	4	12	91	24	13	5	1	1	1

## Footnotes

1 https://uk.mathworks.com/help/driving/ref/multiobjecttracker-system-object.html
